# Neuromodulatory role of L-arginine: nitric oxide precursor against thioacetamide-induced-hepatic encephalopathy in rats via downregulation of NF-κB-mediated apoptosis

**DOI:** 10.1007/s11356-023-28184-7

**Published:** 2023-06-28

**Authors:** Ahmed A. Sedik, Azza Hassan, Dalia O. Saleh

**Affiliations:** 1 Pharmacology Department, Medical Research and Clinical Studies Institute, National Research Centre, Giza, Cairo Egypt; 2 Pathology Department, Faculty of Veterinary Medicine, Cairo University, Giza, Egypt

**Keywords:** L-arginine, Nitric oxide, Thioacetamide, Hepatic encephalopathy, NF-κB, Apoptosis

## Abstract

The aim of the present study was to investigate the impact of arginine (ARG), a nitric oxide (NO) precursor, on thioacetamide (TAA)-induced hepatic encephalopathy (HE) in rats by injection of TAA (100 mg/kg, i.p) three times per week for six consecutive weeks. TAA-injected rats were administered ARG (100 mg/kg; p.o.) concurrently with TAA for the six consecutive weeks. Blood samples were withdrawn, and rats were sacrificed; liver and brain tissues were isolated. Results of the present study demonstrated that ARG administration to TAA-injected rats revealed a restoration in the serum and brain ammonia levels as well as serum aspartate transaminase, alanine transaminase, and alkaline phosphatase and total bilirubin levels as well as behavioral alterations evidenced by restoration in locomotor activity, motor skill performance, and memory impairment. ARG showed also improvement in the hepatic and neuro-biochemical values, pro-inflammatory cytokines, and oxidative stress biomarkers. All these results were confirmed by histopathological evaluation as well as ultrastructural imaging of the cerebellum using a transmission electron microscope. Furthermore, treatment with ARG could ameliorate the immunological reactivity of nuclear factor erythroid-2-related factor 2 (Nrf2) and cleaved caspase-3 proteins in the cerebellum and hepatic tissues. From all the previous results, it can be fulfilled that ARG showed a beneficial role in modulating the adverse complications associated with TAA-induced HE in rats via reducing hyperammonemia and downregulating nuclear factor kappa B (NF-κB)-mediated apoptosis.

## Introduction

Hepatic encephalopathy (HE) is described as a range of neuropsychiatric disorders in patients associated with liver dysfunction (Kabaria et al. 2021). To date, the global acute incidence and occurrence of HE are unknown, possibly due to HE severity, etiological causes. Moreover, it is very difficult to differentiate between mild and subclinical types of HE (López-Franco et al. 2021). However, it is now recognized that HE affects up to 40% of individuals suffering from cirrhosis. Clinical symptomatic features of HE encompass lethargy, lack of cognitive features, depressed consciousness, and coma (Saleh et al. 2021).

Hyperammonemia and inflammation act synergistically to induce the pathogenic events associated with HE, where the elevated ammonia (NH3) levels could freely cross the blood–brain barrier (BBB) leading to impairment in the redox homeostasis (Afifi et al. 2021). Nitric oxide (NO) is a highly reactive prooxidant produced by parenchymal and non-parenchymal cells in the liver, which acts as an important mediator of many physiological and pathophysiological events and commonly triggers the antioxidant response via the transcription factor, nuclear erythroid-2-related factor 2 (Nrf2), which is responsible for the upregulation of the activity of antioxidant enzymes that leads to restoring of cellular redox homeostasis (Cuadrado 2021). The impairment in oxidative homeostasis triggers the nuclear factor kappa B (NF-κB) signaling pathway to augment the secretion of tumor necrosis factor-alpha (TNF-α), and interleukin-6 (IL-6), which are the most critical regulatory signals for initiating liver regeneration (Liang et al. 2022). Thus, the therapeutic utility of reducing the inflammation in the treatment of the cognitive deficits in patients with HE (Cauli et al. 2007) has been extensively proposed to be a novel therapeutic target (Luo et al. 2015).

Meanwhile, there are several clinical trials and drugs that could be valuable for patients with HE, and most of them are predominantly concerned with the reduction of either NH3 production or its absorption. A theory under the theme “assessing with the delivery of essential amino acids” could improve the health life of patients under various pathological conditions and becomes a booming treatment approach (Shao and Hathcock 2008).

L-arginine (ARG), an essential dietary amino acid, is a substrate for NO formation which acts as a neurotransmitter to mediate the production of glutamate, gamma-aminobutyric acid (GABA), and dopamine (Attia et al. 2020). NO stimulates guanylyl cyclase to release cyclic guanosine monophosphate, a mediator of neuronal plasticity and excitability (Biojone et al. 2015). Reduced NO has been linked to cognitive impairment (Olusanya et al. 2018). ARG has been reported to boost NO levels in the prefrontal cortex and hippocampus, which corresponds with cognitive improvement (Wei et al. 2013). Moreover, ARG possesses antioxidant and anti-inflammatory actions by decreasing inflammatory mediators and potentiating the activity of antioxidant enzymes (Liang et al. 2018).

Consequently, the goal of the current study was to evaluate the neuromodulatory role of ARG, a NO precursor with the ability to cross BBB, against HE induced by thioacetamide (TAA) in rats. To highlight the mechanisms behind its coveted neuroprotective function, the present study objective was also extended to investigate ARG role in *induction of Nrf-2, and inhibition of NF-κB*, as crucial regulators of inflammatory signaling and their neurological complications as well as its mediated apoptosis.

To fulfill this objective, elevated serum and brain NH3 levels were estimated that affect the locomotor activity, motor skill performance, and memory impairment. Serum biochemical markers illustrating hepatic injury were monitored. Hepatic and brain damage were evaluated by estimating tissue redox indices and pro-inflammatory cytokines. Our research was further expanded to reveal the histopathological lesions in the liver and cerebellum. Furthermore, the ultrastructural picture of cerebellum using transmission electron microscope was examined. Moreover, with the immune reactivity of Nrf2 and cleaved caspase-3 proteins in with cerebellum and hepatic tissue to validate the involvement of the inflammatory response and apoptosis.

## Materials and methods

### Materials

#### Experimental animals

Male Wistar adult albino rats (8–10 week’s age and weighing 180–200 g) were obtained and housed in a comfortable laboratory setting and fed a standard pellet diet with free access to drinking water under optimum temperature of (23 ± 1 °C) with a 12-h light/12-h dark cycle at the colony unit of the National Research Centre (Egypt). The experimental studies were carried out in accordance with the relevant policies authorized by the National Research Centre’s Medical Research Ethics Committee (MREC) (approval number: 18222).

#### Drugs and chemicals

L-arginine (ARG) was obtained from Merck (CAS no.: 74–79-3), Germany. Thioacetamide (TAA) was purchased from Sigma-Aldrich, USA (CAS no.: 62–55-5). TAA and ARG were freshly prepared in sterile saline.

### Methods

#### Experimental design

Twenty-four male Wistar albino rats were randomly allocated into 3 groups (8 rats/group). Group 1 served as the negative control group and received vehicle (saline) orally throughout the experiment. Group 2 served as the positive control group and received TAA (100 mg/kg, i.p) three times weekly for 6 weeks (Singh et al. 2014, Singh and Trigun 2010). Group 3 is treated with ARG (100 mg/kg, p.o.) concurrently with TAA for six consecutive weeks (Mansour et al. 2002; Trifiletti 1992). The current experimental design was adopted to compare between treated and untreated groups to assess the effect of ARG on TAA-treated rats using statistical methods. Locomotor activity, motor skill performance, and the novel object recognition test (NOR) were assessed before the experimental period and 18 h after the final dose of drugs. Blood samples were taken from the retro-orbital plexus of the eye of rats after being anesthetized with ketamine (100 mg/kg) and xylazine (10 mg/kg) within 24 h from the last administration of the drugs, and the sera were separated by centrifugation at 3000 rpm for 15 min at 4 °C, using a cooling centrifuge (Laborezentrifugen, 2k15, Sigma, Germany). For histological evaluation and immunohistochemical studies, a portion of the liver and brain specimens were kept in 10% formalin.

#### Behavioral assessment

##### Locomotor activity 

Using an activity cage, locomotor activity was assessed based on the infrared photocell principle over a 5-min interval period for each tested rat over a 60-min period (model no. 7420; Ugo Basile, Italy) (Marazioti et al. 2005).

##### Rotarod test

An accelerating rotarod apparatus (model no. 7750; Ugo Basile) was utilized to evaluate the motor skill performance of all rats tested (Vijitruth et al. 2006). Then, the falling latency time was recorded after three training sessions.

##### Novel object recognition test (NOR)

A novel object recognition test (NOR) was designed as described by (Ennaceur and Delacour 1988). Before beginning the test, rats were given 3 days/2 min/each rat to explore the apparatus freely. On the testing day, two trials were conducted: (T1), two identical objects (F) were placed in two opposite corners of the apparatus. In the second experiment (T2), a novel object (N) was used to substitute one of the identical objects from the first experiment (T1), after which the rats were exposed to two different objects: the familiar (F) and the novel (N), and the discriminating index (DI) was calculated. DI is the exploration time difference given as a percentage of the total time spent examining the two items, 1 in T2. DI = N-F/N + F was then calculated.

#### Biochemical assessment

Serum NH3 levels were measured immediately using a colorimetric kit (da Fonseca-Wollheim 1973). Serum levels of aspartate transaminase (AST), alanine transaminase (ALT), alkaline phosphatase (ALP) (Belfield and Goldberg 1971, Reitman and Frankel 1957), and total bilirubin (T. bilirubin) (Walter and Gerarde 1970) were estimated colorimetrically.

Rats were euthanized by decapitation and sacrificed immediately after blood sampling, and liver and brain tissues were quickly washed in ice-cold saline, blotted dry on filter paper, weighed, and stored at 80 °C for future biochemical analysis. One gram of hepatic and brain tissues was homogenized in ice-cold PBS to make a 20% w/v homogenate for measuring brain ammonia levels (Konitzer and Voigt 1963); lipid peroxidation product, malondialdehyde (MDA), was determined by monitoring the thiobarbituric acid reactive substance formation (Ohkawa et al. 1979); and reduced glutathione (GSH) content was determined colorimetrically (Bulaj et al. 1998; Ellman 1959). Nitric oxide (NO) values were evaluated using a colorimetric method based on the Griess reaction (Montgomery and Dymock 1961). Superoxide dismutase (SOD) was measured as the degree of inhibition of auto-oxidation of pyrogallol at an alkaline pH (Marklund and Marklund 1974). Brain content of TNF-α and IL-6 was evaluated in brain homogenate by using the rat TNF-α ELISA kit (Sunlong Biotech Co., Catalog no. SL0722Ra, China) and IL-6 ELISA Kit (Sunlong Biotech Co., Catalog no. SL0411Ra, China), at a wavelength of 450 nm (Sedik and Elgohary 2023). Brain levels of NF-κB (EIAab, catalogue no. SEB824Mu, China) were evaluated according to the manufacturer’s instructions (Khalifa et al. 2023).

#### Histopathological examination

Different hepatic and cerebellum sections were excused and fixed in 10% neutral-buffered formalin to be further stained with H&E (5-µm-thick sections).

#### Immunohistochemical analysis

Immunohistochemical staining of cleaved caspase-3 and Nrf2 in the hepatic and cerebellum tissues of normal and treated rats was carried out (El Awdan et al. 2018). Rat polyclonal anti-caspase-3 (ab13847, Abcam) and rat polycloal anti-Nrf2 antibodies were then used to incubate the sections (ab3113, Abcam). The immune staining was visualized and graded from 0 to 3, according to the percentage of positive cells in random ten microscopic high-power field (40 ×), as follows: 0 = 0 (NO staining), 1 (positive staining in less than 30% of cells/HPF), 2 (30–70% of cells/HPF), or 3 (> 70% of cells/HPF) (Hassan et al. 2019).

#### Electron microscopy of the cerebellum

The dissected cerebellum was fixed in 0.2-M phosphate buffer for 5–6 h at 48 °C with glutaraldehyde. The fixed brains were dissected into 1-mm cubes. The cubes were postfixed for 2 h at 48 °C in 2% OsO_4_ and then dehydrated in an ethaNOl series. The fragments were embedded in EPON/812 TAAB and then sliced into 0.5-mm-thick sections with an ultramicrotome (LKB Sweden) before being mounted on nickel grids (300 mm). The sections were dyed twice with uranyl acetate and lead citrate and then evaluated and photographed using a TEM (Philips CM100, the Netherlands) (Reynolds 1963).

### Statistical analysis

The results are presented as mean ± SEM (8) rats, with all statistical analyses performed using one-way analysis of variance (ANOVA) and Tukey’s multiple comparison test. The statistics were examined using GraphPad Prism v. 8.0. (GraphPad Software, Inc., CA, USA). It is deemed significant when the difference has a *P*-value of ≤ 0.05.

## Results

### Role of ARG on serum hepatic indices in rats received TAA-induced HE in rats

Rats received i.p. dose of TAA (100 mg/kg) three times weekly for six consecutive weeks showed an increase in serum NH_3_, AST, ALT, ALP, and T. bilirubin levels nearly 141%, 137%, 214%, 443%, and 133% of the normal value, respectively. Serum levels of NH_3_, AST, ALT, ALP, and T. bilirubin values were reduced by roughly 102%, 103%, 101%, 104%, and 101% of the normal value, respectively, after oral treatment of HE rats with ARG (100 mg/kg) for six consecutive weeks as compared to TAA group (Table 1).

**Table 1:** Role of ARG on serum biochemical parameters in rats received TAA-induced HE

Parameter/group	Serum NH_3_ (µmol/L)	AST (IU/L)	ALT (IU/L)	ALP (IU/L)	T. bilirubin (mg/dl)
Normal	**87.90 ± 0.44**	**243.7 ± 0.89**	**90.21 ± 0.69**	**192.6 ± 0.81**	**0. 44 ± 0.02**
TAA (100 mg/kg)	123.8 ± 0.92*	322.8 ± 1.5*	123.4 ± 0.97*	411.8 ± 0.76*	1.950 ± 0.01*
AG (100 mg/kg)	**90.43 ± 0.08@**	**246.5 ± 0.66@**	**92.79 ± 0.31@**	**195.3 ± 0.54@**	**0.46 ± 0.02@**

IP injection of TAA at 100 mg/kg thrice weekly for 6 weeks with was used to induce HE. Oral treatment of TAA induced-HE with ARG (100 mg/kg). Twenty-four hours after the last dose of the drugs, serum levels of NH3, AST, ALT, ALP, and T. bilirubin were evaluated. Results are expressed as mean ± SEM (*n* = 8). *Significant difference from normal control group *p* < 0.05. @Significant difference from TAA-received group

### Role of ARG on locomotor activity, motor skill performance, and NOR in rats received TAA-induced HE

**Fig. 1:**
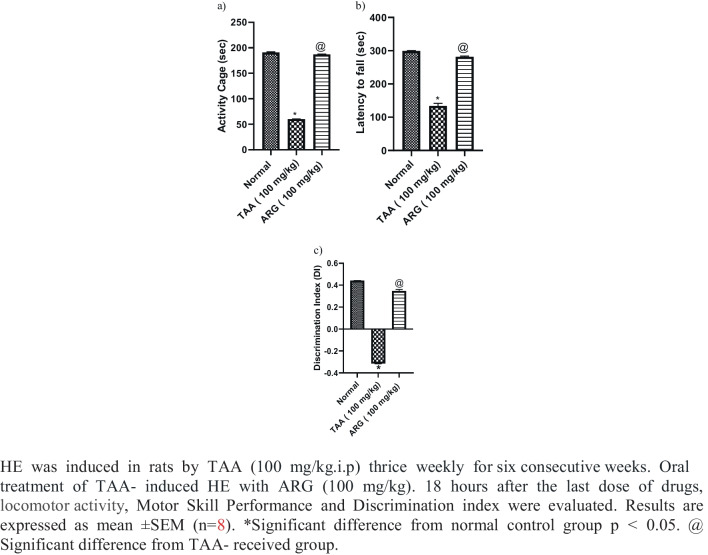
Role of ARG on locomotor activity, motor skill performance, and NOR in rats received TAA-induced HE

TAA-treated rats exhibited a decrease in locomotor activity and motor skill performance of about 32% and 44% of normal values, respectively. Oral administration of ARG (100 mg/kg) to HE rats for 6 weeks in conjunction with TAA could normalize the previously described parameters (Fig. 1a and b). Regarding ORT, TAA-treated rats showed an inability to distinguish between familiar and novel items. The novel item was considerably distinguished from the familiar object after 6 weeks of oral treatment of ARG (100 mg/kg) to HE rats in conjunction with TAA (Fig. 1c).

### Role of ARG on hepatic and brain oxidative stress indices in rats received TAA-induced HE

HE was associated with a decrease in hepatic and brain GSH levels of about 42% and 45% of normal values, respectively, and a rise in hepatic and brain MDA levels of about 152%t and 153% of normal values, respectively. Oral treatment of HE rats with ARG (100 mg/kg) in combination with TAA for 6 weeks increased hepatic and brain GSH levels that were 96% and 94% of normal values, respectively, as well as a normalized value in hepatic and brain MDA levels as compared with TAA group (Table 2).

**Table 2:** Role of ARG on GSH, MDA, and NO levels and SOD activity in hepatic and brain homogenate of rats received TAA-induced HE

Parameter/group	GSH levels (mg/g)		MDA (nmol/g)		NO (nmol/g)		SOD activity (U/mg tissue)	
	Liver	Brain	Liver	Brain	Liver	Brain	Liver	Brain
Normal	**51.24 ± 0.49**	**31.28 ± 0.37**	**139.5 ± 0.28**	**90.25 ± 0.2**	**71.67 ± 0.33**	**49.27 ± 0.83**	**1.87 ± 0.01**	**1.75 ± 0.01**
TAA (100 mg/kg)	21.50 ± 0.65*	14.06 ± 0.42*	213 ± 0.41*	135 ± 0.9*	98.85 ± 0.42*	68.83 ± 0.78*	1.34 ± 0.03*	1.43 ± 0.01*
ARG (100 mg/kg)	**49.60 ± 0.19@**	**29.67 ± 0.33@**	**141 ± 0.45@**	**88.46 ± 0.2@**	**72.21 ± 0.58@**	**45.58 ± 1.91@**	**1.82 ± 0.03@**	**1.79 ± 0.06@**

IP injection of TAA at 100 mg/kg thrice weekly for 6 weeks which was used to induce HE. Oral treatment of TAA-induced HE with ARG (100 mg/kg). Twenty-four hours after the last dose of the drugs, hepatic and brain levels of GSH, MDA, NO, and SOD were evaluated. Results are expressed as mean ± SEM (*n* = 8). *Significant difference from normal control group *p* < 0.05. @Significant difference from TAA-received group

Intraperitoneal injections of TAA at 100 mg/kg three times a week for 6 weeks resulted in a 72% and 82% drop in hepatic and brain SOD levels, respectively, compared to the normal value. For 6 weeks, HE rats were given ARG (100 mg/kg) together with TAA, and their hepatic and brain MDA levels were adjusted (Table 2).

### Role of ARG on hepatic and brain NO levels in rats received TAA-induced HE in rats

HE was accompanied by a marked increase in hepatic and brain NO levels of around 137% and 139% of normal values, respectively. The hepatic and brain NO levels in HE rats were reduced by around 101% and 92%, respectively, after 6 weeks of oral therapy with ARG (100 mg/kg) in combination with TAA (Table 2).

### Role of ARG on brain NH3, NF-κB signaling pathway, and pro-inflammatory cytokines: TNF-α and IL-6 in the brain of rats received TAA-induced HE in rats

**Fig. 2:**
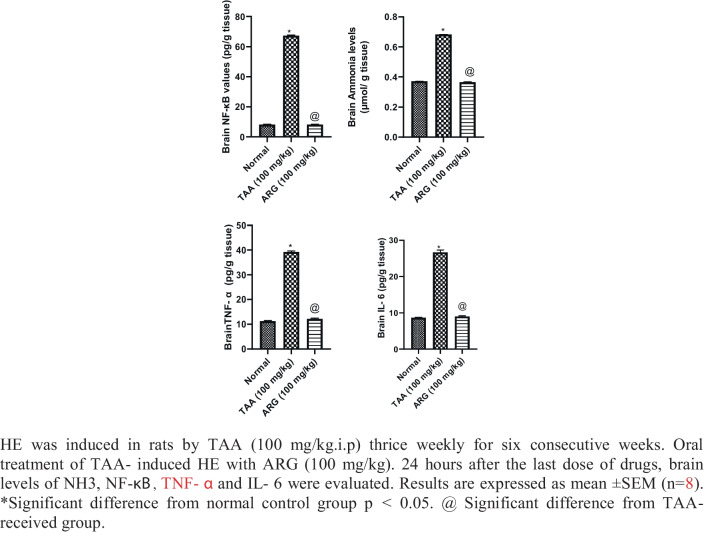
Role of ARG on brain NF-κb, NH3, TNF-α, and IL-6 levels in rats received TAA-induced HE

Brain NH3, NF-κB, TNF-α, and IL-6 levels increased roughly 183%, 8-, 3-, and three-folds of the normal value, respectively, in TAA-injected rats. Oral treatment of HE rats with ARG (100 mg/kg) for 6 weeks was successful in normalizing the parameters (Fig. 2a, b, c, d).

### Role of ARG on the cerebellum of rats received TAA-induced HE as observed under histopathological examination

**Fig. 3:**
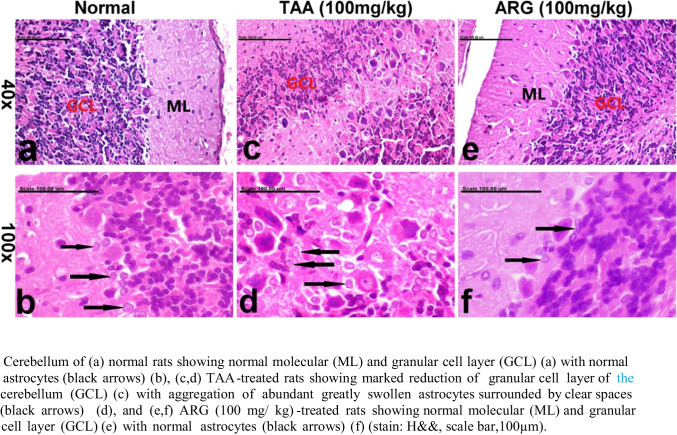
Role of ARG on cerebellum in TAA-induced HE in rats as observed under histopathological examination

The cerebellum of (a) normal rats revealed normal granular and molecular cell layer (a) with normal astrocytes (b, c, and d), as shown in Fig. 3(a, b, c, d). Meanwhile, cerebella of rats that received TAA showed an obvious reduction in the granular cell layer of the cerebellum (c) with aggregation of abundant greatly swollen astrocytes surrounded by clear spaces (d) and (e and f). Conversely, the cerebellum of rats that received ARG (100 mg/ kg) showed normal cerebellum (e) with normal astrocytes (f) (stain: H&E, scale bar, 100 µm) (Fig. 3e and f).

### Role of ARG on hepatic tissue of rats received TAA-induced HE as observed under histopathological examination

**Fig. 4:**
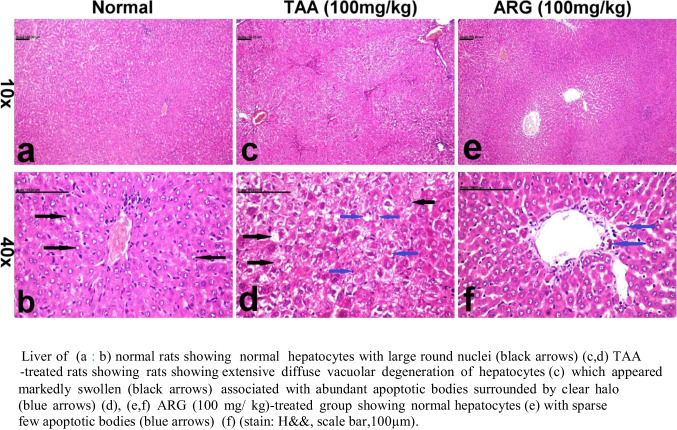
Role of ARG on hepatic tissue in TAA-induced HE in rats as observed under histopathological examination

Liver of (a and b) normal rats showed normal hepatic structure (c and d). Meanwhile, rats received TAA revealed extensive diffuse vacuolar degeneration of hepatocytes (c) which appeared markedly swollen and associated with abundant apoptotic bodies surrounded by a clear halo (d, e, and f). Normal hepatocytes (e) with sparse few apoptotic bodies (f) were observed in ARG group (100 mg/kg) (stain: H&E, scale bar, 100 µm) (Fig. 4).

### Role of ARG on Nrf2 and caspase-3 expression in the cerebellum of rats received TAA-induced HE

Table 3 revealed the representative values for the expression of cleaved caspase-3 and Nrf2 levels in the cerebellum of normal rats and others received either TAA or ARG.

**Table 3:** Role of ARG on the expression of cleaved caspase-3 and Nrf2 values in the liver and cerebellum in rats treated with TAA-induced HE

Parameter/group	Cleaved caspase-3 (% of positive cells/HPF)		Nrf2 (% of positive cells/HPF)	
	Liver	Cerebellum	Liver	Cerebellum
Normal	**0.00 ± 0.00**	**0.00 ± 0.00**	**1.10 ± 0.10**	**1.30 ± 0.15**
TAA (100 mg/kg)	2.90 ± 0.10*	2.50 ± 0.10*	0.30 ± 0.15*	0.30 ± 0.10*
ARG (100 mg/kg)	1.30 ± 0.16*@	1.40 ± 0.26*@	2.40 ± 0.16*@	2.00 ± 0.21*@

IP injection of TAA at 100 mg/kg thrice weekly for 6 weeks which was used to induce HE. Oral treatment of TAA-induced HE with ARG (100 mg/kg). Twenty-four hours after the last dose of the drugs, cerebellum and hepatic levels of caspase-3 and Nrf2 were evaluated. Results are expressed as mean ± SEM (*n* = 8). *Significant difference from normal control group *p* < 0.05. @Significant difference from TAA-injected group

**Fig. 5:**
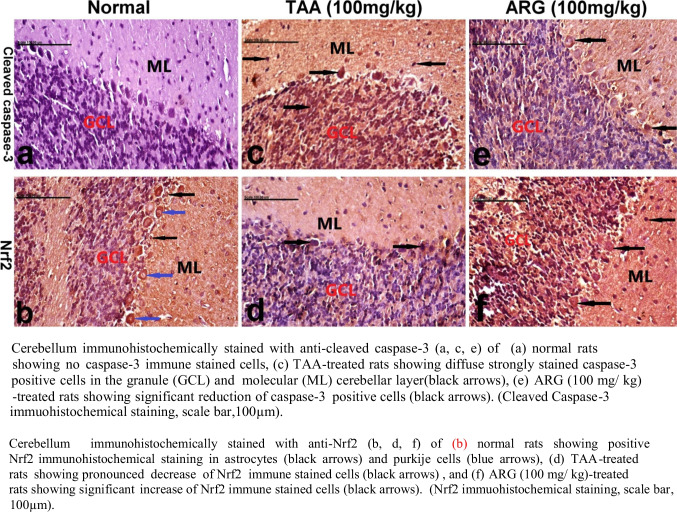
Role of ARG on cleaved Caspase-3 and Nrf2 expression in the cerebellum of TAA-induced HE in rats

Cerebellum immunohistochemically stained with *anti-cleaved caspase-3* (a, c, and e) of (a) normal rats revealed NO caspase-3 immune stained cells; (c) TAA-treated rats showed diffuse strongly stained caspase-3-positive cells in the granule and molecular cerebellar layer. A significant increase of caspase-3-positive cells (e) was recorded in rats which received ARG (100 mg/kg), scale bar, 100 µm (Fig. 4). Meanwhile, cerebellum immunohistochemically stained with *anti-Nrf2* (b, d, and f) of (a) normal rats showing positive Nrf2 immunohistochemical staining in astrocytes and Purkinje cells and (d) TAA-treated rats showing marked decline in Nrf2 immune stained cells. Significant increase of Nrf2 immune stained cells (f) was recorded in rats that received ARG (100 mg/ kg), scale bar, 100 µm (Fig. 5).

### Role of ARG on Nrf2 and caspase-3 expression in hepatic tissue of rats received TAA- induced HE

Table 3 showed the representative values for the expression of cleaved caspase-3 and Nrf2 levels in the liver of normal rats and rats received either TAA or ARG.

**Fig. 6:**
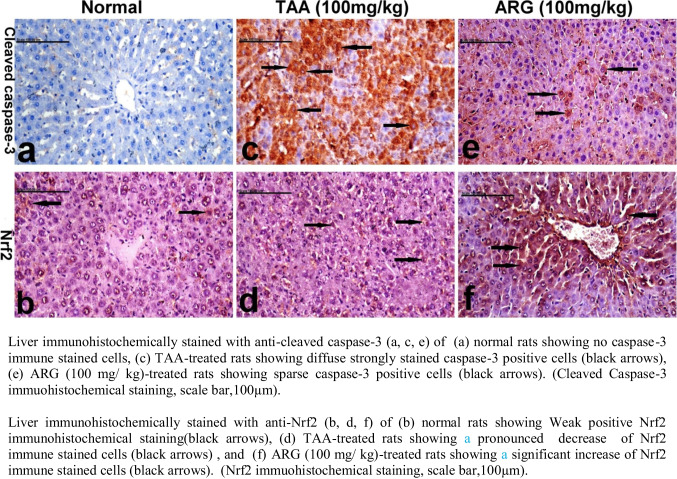
Role of ARG on cleaved Caspase-3 and Nrf2 expression in the liver of TAA-induced HE in rats

Liver immunohistochemically stained with *anti-cleaved caspase-3* (a, c, e) of (a) normal rats revealed NO caspase-3 immune stained cells, (c) TAA-treated rats showed diffuse strongly stained caspase-3-positive cells, and (e) ARG (100 mg/kg)-treated rats showed sparse caspase-3-positive cells, scale bar, 100 µm (Fig. 5). Meanwhile, liver immunohistochemically stained with *anti-Nrf2* (b, d, f) of (b) normal rats showed weak-positive Nrf2 immunohistochemical staining, (d) TAA-treated rats revealed pronounced decrease of Nrf2 immune stained cells, and (f) ARG + TAA (100 mg/g)-treated rats showed significant increase of Nrf2 immune stained cells, scale bar, 100 µm (Fig. 6).

### Role of ARG on the cerebellum of rats received TAA-induced HE as observed under TEM

**Fig. 7:**
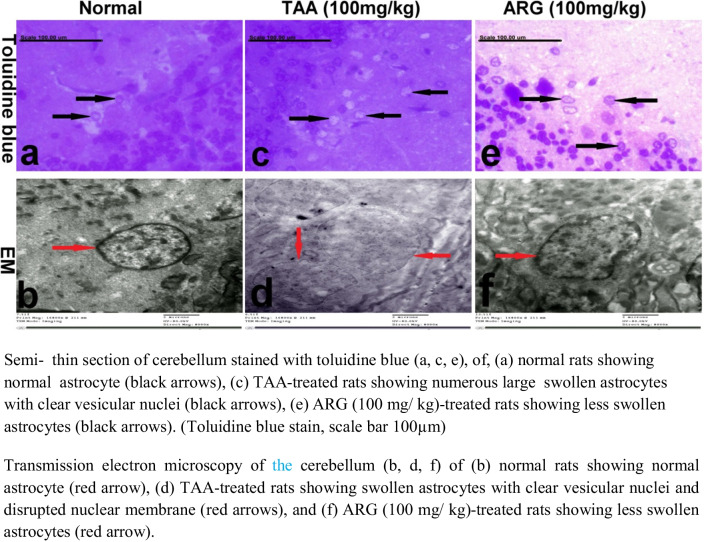
Role of ARG on the cerebellum of rats received TAA-induced HE as observed under TEM

The cerebellum of nornal rats stained with toluidine blue showed normal astrocytes with an intact nuclear membrane in TEM (Fig. 7a and b), whereas a significant number of swollen astrocytes with vesicular nuclei were observed in TAA-received group (Figs. 7c and d). On the other hand, astrocyte swelling was significantly decreased in ARG-treated group (Fig. 7e and f).

## Discussion

Hepatic encephalopathy is a serious neuropsychiatric complication that can develop in people with hyperammonemia, which is usually caused by cirrhosis, liver failure, and some circulatory anomalies caused by portosystemic shunting. (Engelmann et al. 2021). However, there have been reports of many non-hepatic causes of hyperammonemia (Ali and Nagalli 2022). In our study, intraperitoneal injection of TAA at a dose of 100 mg/kg, three times per week for six consecutive weeks, was selected as a model of HE to mimic the pathophysiological events associated with human HE, starting from increased ammonia levels, oxidative stress imbalance evidenced by elevation in MDA, and reduction in GSH and SOD levels indicating excessive production of reactive oxygen species (ROS) with an elevation lipid peroxidation in brain cells. This eventually elevate the levels of pro-inflammatory cytokines and thus cause apoptosis (Hajipour et al. 2021).

Hepatic encephalopathy is typically associated with disturbances in motor and cognitive functions ranging from sleep disturbances to severe cognitive and motor impairment (Volkow et al. 1998). Our study revealed that inducing HE with TAA had a significant decline in locomotor activity, which is a clinical parameter for determining the severity of HE (Ahboucha et al. 2008). In support of this result, it has been documented that TAA-induced fulminant HE was related with an impairment in locomotor coordination. (Leke et al. 2012). Similarly, rats received TAA significantly showed a decrease in motor skill performance in experimental rats causing motor and learning impairment because of neuroinflammation associated with massive increment in NH3 levels (Méndez et al. 2008). Oral dosing of ARG (100 mg/kg) in conjunction with TAA for six consecutive weeks revealed a marked improvement in cognitive and motor functions because of its role as a NO donor especially in the cerebellum that is responsible for the control of motor and cognitive movements (Yi et al. 2009). Our study agreed with a previous study that revealed that ARG had a marked positive role in growing motor and cognitive activities especially at the prefrontal cortex and hippocampus, both areas which are important for cognitive improvement (Mahmoud et al. 2021).

Additionally, our findings stated that TAA-induced HE in rats was associated with memory impairment and a distorted ability to differentiate between the novel and familiar objects manifested by NOR due to over production of peroxynitrite radicals within the cerebellum inducing memory and learning impairment (Baliou et al. 2021). Concurrent dosing of TAA-injected rats with ARG resulted in a significant improvement in non-spatial working memory, as rats detected the new object more than the familiar one (Angelova et al. 2021; Orzelska et al. 2015).

Hyperammonemia is a life-threatening detrimental factor in the incidence and progression of HE as a nitrogenous substance especially NH3 could rapidly accumulate in the circulation and freely crosses BBB to the brain to cause astrocyte enlargement and cytotoxic edema in the brain (Ismail et al. 2021). HE caused by TAA was linked to impairment in urea synthesis and an increase in serum NH3 levels with a remarkable increase also in the brain. Treated rats with ARG significantly decrease the serum and brain NH3 levels due to the marked action of ARG on the gut to prevent the conversion of NH3 into ammonium be trapped into the gut and never crosses BBB (Schaefer et al. 2002).

Our current findings revealed that rats received TAA at a dose of 100 mg/kg i.p. three times weekly for 6 weeks had elevated levels of ALT, AST, and ALP activities with a remarkable elevation in the concentration of bilirubin (Hao et al. 2011). These results were allied with a previous study (Semwal et al. 2016). The authors reported that increased levels of hepatic enzymes due to TAA administration implies cellular leakage and loss of functional integrity of the hepatic cell membrane (Mansour et al. 2017). In addition, the administration of TAA was associated with excessive bilirubin levels due to a significant release of ROS leads to pronounced destruction of hepatic cells (Yin et al. 2007). Oral administration of rats received TAA with ARG for six consecutive weeks revealed marked amelioration in the levels of serum hepatobiliary enzymes toward normal levels which is attributed to capacity of ARG to protect plasma membranes from the outbreak of free radicals and prevent their damage (Tripathi and Misra 2009).

Following the previous results, there are two crucial factors for initiation and development of HE which are inflammation and oxidative stress. Many studies revealed that hepatic inflammation frequently occurs within a few hours of TAA injection. Therefore, there is no doubt that oxidative damage and hepatic inflammation are corrected in the course of liver damage (Seyan et al. 2010). Previous findings found that oxidative stress and inflammation are inextricably linked due to initiating secretion and production of NF-κB-mediated pro-inflammatory mediators (Kang et al. 2013). NF-κB is a critical transcriptional regulator of the inflammatory response, regulating many functions such as immunological responses and cell survival in hepatocytes Kupffer cells and hepatic stellate cells (Taniguchi and Karin 2018). In the current work, ARG significantly reduced TNF-α and IL-6 levels though inhibiting NF-*κ*B activation in rats, implying that the anti-inflammatory action may be one potential strategy against TAA-induced HE (Develi‐Is et al. 2013).

The effect of ARG on nuclear Nrf2 levels has been evaluated in the liver and the cerebellum of rats to acquire a better understanding of the probable antioxidant mechanism of ARG. Nrf-2 is responsible for the upregulation of the activity of antioxidant enzymes that lead to restoring of cellular redox homeostasis (Hejazian et al. 2021). Indeed, several findings confirmed that TAA is strongly linked with significant decline in Nrf-2 activity (Alsheblak et al. 2016). Our findings showed that the levels of nuclear Nrf-2 in the liver and cerebellum of rats given TAA were significantly decreased. However, ARG treatment restored Nrf2 expression to the normal levels. It is possible that ARG conserved NH3 concentration in liver tissue by acting as an antioxidant and lowering hepatic NO overload, resulting in an extensive hepatoprotective effect (Radwan 2012).

Growing evidence indicates that NO produced by inducible nitric oxide synthase (iNOS) plays a pathogenic role in the brain (Kröncke et al. 1998). NO shares critically in the development of HE because increased NO production in the brain causes cerebral vasodilation, which increases capillary surface area and allows NH3 to diffuse easily (Mostafa et al. 2017). Hyperammonemia, on the other hand, is associated with enhanced BBB permeability to pro-inflammatory cytokines to facilitate the production of iNOS. The large increase in NO levels in the liver and brain of rats given TAA could be attributable to the neuroinflammatory interaction between NO and superoxide, which produces peroxynitrite radicals, according to our findings (Milewski et al. 2021). This conclusion was consistent with earlier studies that found a link between iNOS overexpression and brain impairment (Yamada et al. 1999). ARG inhibits NOS gene expression and enzyme activity by acting on the NF-κB sites in the iNOS promoter, resulting in iNOS gene downregulation, and by directly inhibiting the catalytic activity of iNOS (Chaturvedi et al. 2010).

The nervous system is largely prone to oxidative damage because the brain is enriched with polyunsaturated fatty acids with a weakness in the defense mechanism (Öztürk et al. 2008). Cells are equipped with endogenous enzymatic or nonenzymatic antioxidants that are central in either slowing or preventing the progression and incidence of pathogenic diseases (Wagener et al. 2013). In the current study, rats received TAA for six consecutive weeks showed a decline in GSH content and the activity of SOD, revealing an impairment occurrence in the antioxidant mechanism in the liver (Dwivedi et al. 2020). Previous studies confirmed that cellular oxidative damage plays a direct role in apoptosis activation. Overproduction of oxidizing species causes mitochondrial malfunction, including loss of mitochondrial membrane potential, release of cytochrome c from the mitochondria into the cytosol, and activation of caspase-3 and apoptosis (El-Maadawy et al. 2021). Caspase-3, the primary executor of apoptosis, has been identified as a prominent strategy for detecting hepatic damage and has been linked to dynamic liver fibrosis (Bourbonnais et al. 2012). In the present study, HE rats that received TAA had an elevated level of caspase-3 due to elevated oxidative stress indices and activation of NF-κB pathway which agreed with previous studies (Furtado et al. 2012, Shin and Lee 2022). Meanwhile, rats treated with ARG showed a significant decline in the expression of caspase-3. This confirmed the neuroprotective role of ARG through stopping the production of ROS, induction of Nrf-2, and inhibition of NF-κB-mediated apoptosis in HE rats (Vervaecke 2017).

Fine structure of the cerebellum and liver of rats that received TAA-induced HE revealed histopathological alterations, abundant greatly swollen astrocytes surrounded by clear spaces with extensive vacuolization, and degeneration of hepatocytes. Inconsistent with prior findings (Shin et al. 2021), the pathogenic alterations in ARG-treated rats were significantly reduced, as revealed by normal astrocytes and normal hepatocytes with scant apoptotic bodies (El Husseiny et al. 2017). Furthermore, electron microscopic results of the cerebellum of HE rats revealed exhibited a significant number of swollen astrocytes with vesicular nuclei due to the deleterious effect of TAA and excessive attack of ROS (Grzybicki 1995). Meanwhile, HE rats treated with ARG had normal astrocyte size and cell membranes, which can be related to ARG’s antioxidant activity and its ability to minimize the hepatic NO overload, preserving NH3 concentration in liver tissue and extended to reduce it to a minimum in the cerebellum (Stevens et al. 1996).

## Conclusion

**Fig. 8:**
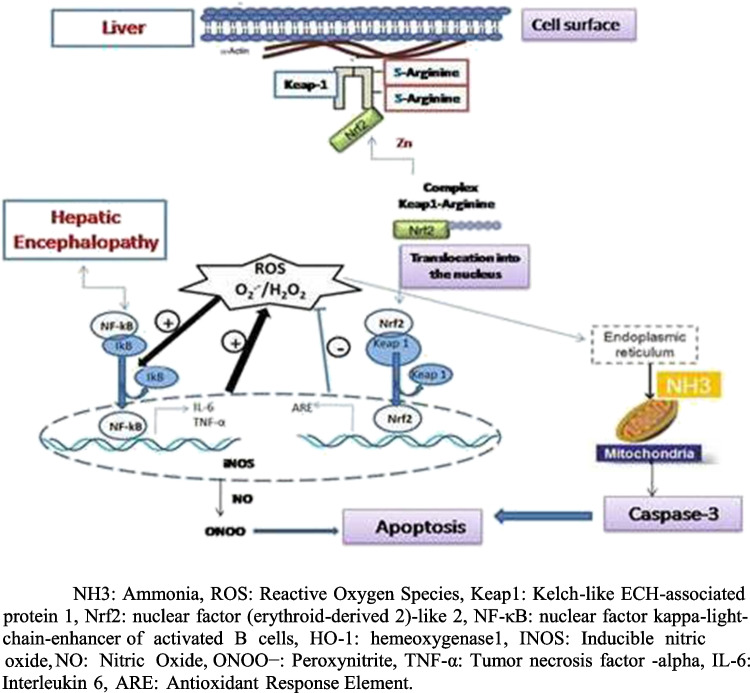
Proposed mechanism of action of arginine on hepatic encephalopathy in rats

According to the previous results, it can be concluded that ARG exhibited a potent hepatoprotective activity along with the effective neuroprotective potential that significantly hindered the development of HE by TAA. As shown in the Fig. 8, our findings documented the significant antioxidant and anti-inflammatory properties of ARG in HE rats. In the NO precursor, ARG was effective in minimizing the deleterious alterations associated with HE via reducing hyperammonemia and potentiating the antioxidant response via inhibition of oxidative stress and thus induce Nrf-2, and inhibit NF-κB-mediated apoptosis and thus inhibited its neurological complications. In addition, its anti-inflammatory effect therefore, this study depicts that ARG exerted new intervention for HE.

## Data Availability

Available upon request.
